# Identification and genomic analysis of two novel duck-origin GPV-related parvovirus in China

**DOI:** 10.1186/s12917-019-1833-9

**Published:** 2019-03-12

**Authors:** Guozhi Bian, Haibin Ma, Mengping Luo, Fengping Gong, Bo Li, Guiping Wang, Mudassar Mohiuddin, Ming Liao, Jianfeng Yuan

**Affiliations:** 1 Veterinary Medicine College of South China Agricultural University, Guangzhou, 510642 China; 2 Guangdong Haid Institute of Animal Husbandry & Veterinary, Guangzhou, 511400 China

**Keywords:** Novel parvovirus-related virus, Phylogenetic analysis, Mule duck, Cherry valley duck

## Abstract

**Background:**

Since early 2015, mule duck and Cherry Valley duck flocks have been suffering from short beak and dwarfism syndrome. This widely spreading infectious disease is characterized by growth retardation, smaller beak and tarsus with high morbidity and low mortality rate. For better understanding, we identified and characterized virus isolates named AH and GD from diseased Cherry Valley duck and mule duck flocks and investigated the damage caused by novel parvovirus-related virus (NGPV) to tissues and organs, including kidney, brain, pancreas, liver, spleen, bursa of fabricius and myocardial tissues.

**Results:**

AH and GD isolates shared high nucleotide identity with goose parvovirus (GPV). Alignment studies of AH and GD isolates showed 94.5–99.2% identity with novel parvovirus-related virus (NGPV), 98.7–91.5% identity with GPV and 79.9–83.7% with muscovy duck parvovirus (MDPV). Compared with other NGPV, classical GPV and MDPV sequences, a four 14-nucleotide-pair insertion in GD isolate was found in left open reading frame (ORF) (87–100 nt and 350–363 nt) and in right ORF (4847–4861 nt and 5122–5135 nt). However, in AH isolate, a five 14-nucleotide-pair deletions similar to other NGPV were found. The complete genome sequence comparison of eleven NGPV isolates from mule ducks and cherry valley ducks revealed no remarkable difference between them. Notably, the myocardium and bursa of fabricius of both disease and healthy animals are perfectly normal while other tissues have inflammatory cells exudation.

**Conclusions:**

The AH and GD strains are novel parvovirus-related virus that isolates from mule ducks or cherry valley ducks which DNA sequence has no remarkable difference. The histopathology of tissues and organs such as kidney, brain etc. revealed non-significant changes in experimental and control animals. Overall, this study has contributed better understanding of molecular biology of NGPV strains and will help to develop the candidate strain for vaccine preparation to get better protection against these viral infections.

## Background

Muscovy duck parvovirus (MDPV) and Goose parvovirus (GPV) are members of the family *Parvoviridae* [1, 2]*.* GPV are etiological agents for Derzsy’s disease and results in huge economic losses to the duck industry [3]. The mortality rate in goslings during first four weeks of life ranges from 70 to 100% [4]. The disease in infected animals is characterized by lethargy, stunting, anorexia, locomotor dysfunction, watery diarrhea and death within 3 to 5 days after disease onset [1, 5, 6]. MDPV infection mainly affects three-week-old Muscovy ducklings and is characterized by signs almost similar to GPV. The complete nucleotide sequence of the two parvoviruses shares 79.7 to 85.0% homology.

Waterfowl parvoviruses, including MDPV and GPV, contain a linear, single-stranded DNA genome (approx. 5.0–5.1 kb in length) [7]. At both ends of GPV and MDPV genome, there are two inverted terminal repeats (ITR) forming a hairpin structure which contains a stem region and a bubble region having length 456 and 442 nucleotides for MDPV and GPV respectively [8, 9]. There are two open reading frames (ORFs) in waterfowl parvoviruses [10, 11]. The left ORF encodes non-structural (NS) proteins NS1 and NS2 and right ORF encodes structural proteins namely VP1, VP2 and VP3 [12, 13].

During 1970s, Short beak and dwarfism syndrome (SBDS) was first reported in France in mule ducks, caused by a novel parvovirus-related virus (NGPV) [14, 15]. Since March 2015, there are numerous SBDS outbreaks in mule duck and Cherry Valley duck flocks in various regions of China [16, 17]. The morbidity rate of NGPV was found to be 10–30% while mortality rate was about 2% [18]. The ducks infected with NGPV, showed clinical symptoms like swollen tongue, shorter tibia and stunted growth [14]. In this way, the disease causes huge economic losses to duck industry by reduction in weight and size of animals. However, the adult ducks were found resistant to SBDS disease [14].

In order to have better understanding of SBDS disease, we characterized two NGPV namely AH and GD isolates, obtained from mule duck and Cherry Valley duck flocks in Anhui and Guangdong province, China. For detailed analysis, the complete genome of these isolates was sequencedand homology studies and phylogenetic comparison was carried out. The nucleotide sequences of the AH and GD genome and ITR were aligned for comparison with other GPV/MDPV/NGPV strains. The amino acid sequences of non-structural and structural proteins were also subjected to phylogenetic and homology analysis. The histopathological features were also studied in experimental and control groups.

## Methods

### Sample collection

In 2014–2015, two mule duck and Cherry Valley duck flocks located in Guangdong and Anhui province, China were found to be suffering from SBDS disease. Liver and spleen samples were collected from diseased birds and processed for viral isolation.

### Virus isolation

The AH and GD strains of variant GPV were isolated from mule duck and Cherry Valley duck flocks with typical signs of SBDS. Liver and spleen tissues were grinded and homogenized in physiological saline, freeze-thawed three times and centrifuged at 10,000 x g for 15 min at 4 °C. The supernatants were subjected to filtration using 0.22 μm sterile filters to remove contaminants and inoculated into chorioallantoic cavity of 10-day-old duck embryonated eggs (200 μl/egg). The allantoic fluid was harvested 5 days after inoculation and stored at − 80 °C for further testing.

### DNA extraction

The allantoic fulid of AH and GD isolates was centrifuged at 5000 xg for 10 min to remove cellular debris and proceeded for DNA extraction using AxyPrep DNA Extraction Kit (ANYGEN). The stock DNA was dissolved in TE buffer for future use.

### Amplification of AH and GD complete genome

To amplify the complete genome of GD and AH isolates, six primer pairs were designed, based on the conserved regions in the genome sequence of NGPV (Table 1). The polymerase chain reaction conditions were as follows: 95 °C for 5 min, followed by 35 cycles of denaturation (95 °C for 30 s), annealing (52–60 °C for 30 s), extension (72 °Cfor 2 min) and a final extension at 72 °C for 10 min. The complete genome sequence was obtained by assembling all six DNA fragments.

**Table 1:** Primers used to amplify the complete genome

Primers	Sequences	Position	Size of PCR products (bp)	Annealing temperature
P1-F	CTCATTGGAGGGTTCGTTCG	1–20	191	58 °C
P1-R	GCATGCGCGTGGTCAACCTAACA	169–191		
P2-F	CAAACGGGGAGGGCAAAATAAGA	186–208	1345	60 °C
P2-R	GTGGTCGCAGGTCCGTAGAGC	1511–1531		
P3-F	GGGTGAAGAGAGAGTTCAACAA	1474–1497	1137	52 °C
P3-R	CGTTACCAGGCCCAAGATAC	2591–2610		
P4-F	GGCATTTGAAAGCTGGAGCC	2473–2492	922	60 °C
P4-R	GTCTTCGTCTGATCCTGCGT	3375–3395		
P5-F	TTGCGATTCCCAATGGATG	3089–3107	1171	58 °C
P5-R	CCCAAATAGGTCCCTGTAGATA	4239–4260		
P6-F	CTACAACCCGGACCTGTGTC	3948–3967	921	60 °C
P6-R	GCATGCGCGTGGTCAACCTAACA	4846–4868		

### Cloning and sequencing

The amplified PCR products were purified using gel extraction kit (AXYGEN), and all fragments were cloned into pMD18-T vector (TaKaRa). The plasmids were sequenced by Sangon Tech (Sangon Biotech, shanghai, China). For ensuring accuracy of genomic sequences, triplicate clones of fragments were sequenced.

### Sequence analysis

Using BLAST sequence alignment, all six fragments were assembled to form a full–length complete genome. The sequence alignment and homology studies for whole-genome, NS (non–structural proteins) and VP (structural proteins) of GD and AH isolates was done by comparison with already reported sequences in the Genbank using MegAlign and MEGA 5.0 software programs [19] (Table 2).

**Table 2:** Parvovirus complete genome sequences used in this study

Virus	Strain	Location	Host	Genome size	Genbank accession no.	Year
Classical GPV	B	Hungary	goose	5106	U25749	1995
Classical GPV	E	China	goose	5125	KC184133	2013
Classical GPV	GDaGPV	China	goose	5106	HQ891825	1978
Classical GPV	SH	China	goose	5106	JF333590	2009
Classical GPV	SYG61v	China	goose	5102	KC996729	1961
Classical GPV	VG32/1	Taiwan	goose	5104	EU583392	2008
Classical GPV	Y	China	goose	5106	KC178571	2011
Classical GPV	YZ99–6	China	goose	5046	KC996730	1999
Classical GPV	LH	China	goose	5047	KM272560	2012
Classical GPV	06–0329	Taiwan	goose	5054	EU583391	2006
Classical GPV	82-0321v	Taiwan	goose	4980	EU582289	2008
MDPV	P	China	Muscovy duck	5123	KU844281	1988
MDPV	GX5	China	Muscovy duck	5132	KM093740	2011
MDPV	FZ91–30	China	Muscovy duck	5131	KT865605	1991
MDPV	SAAS-SHNH	China	Muscovy duck	5061	KC171936	2013
NGPV	SDLC01	China	Cherry Valley duck	5006	KT343252	2015
NGPV	QH15	China	Cherry Valley duck	5048	KT751090	2015
NGPV	SDLY1512	China	Cherry Valley duck	5054	MF441221	2015
NGPV	SDLY1604	China	Cherry Valley duck	5054	MF441223	2016
NGPV	SDLY1605	China	Cherry Valley duck	5054	MF441224	2016
NGPV	AH1606	China	Cherry Valley duck	5054	MF441225	2016
NGPV	JS1603	China	Cherry Valley duck	5055	MF441226	2016
NGPV	AH	China	Cherry Valley duck	5053	MH444513	2015
NGPV	GD	China	mule duck	5106	MH444514	2016
NGPV	M15	China	mule duck	5030	KU844283	2015
NGPV	MDE	China	mule duck	5106	MF438102	2015

### Histopathological examination

Histopathological investigations were carried out in the diseased flocks by comparison with the healthy (Control) animals. At 14 days post infection (DPI), the animals were euthanized and necropsied. Intravenous anesthesia of high concentrations of pentobarbital sodium (150 mg/kg) was used in this study. Samples of kidney, brain, pancreas, liver, spleen, bursa of fabricius and myocardial tissues were fixed with 10% formalin; all the tissues were dehydrated, and immersed in transparent wax, cut into slices and stained with H & E dye. All of the animal protocols were performed in accordance with the ‘Guidelines for Experimental Animals’ of Ministry of Science and Technology (Beijing, China).

## Results

### Viral isolation

**Fig. 1:**
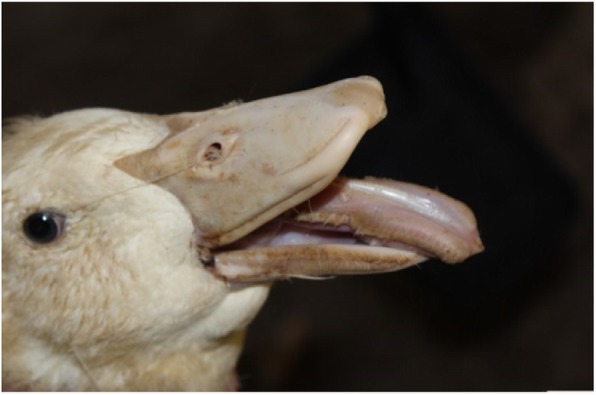
Clinical signs in infected ducklings. The main signs and symptoms in AH or GD-infected ducklings were retarded in growth

Two NGPV strains (GD and AH) were isolated from NGPV-positive mule duck and Cherry Valley duck samples respectively. These isolates were not able to cause death in duck embryonic eggs 5 days post infection (dpi). The main signs and symptoms in AH or GD-infected ducklings were retarded in growth with low mortality rate (Fig. 1).

### Complete genome sequence analysis

The complete genome sequence of mule duck isolate “GD” and Cherry Valley duck isolate “AH” was 5106 bp and 5053 bp respectively. The sequences were submitted to GenBank and their accession numbers are indicated in Table 2. The NGPV genome contained two identical inverted terminal repeats (ITRs) and two open reading frames (ORFs). The length of ITRs of AH and GD genome was 379 and 407 nucleotides (nt) respectively.

**Fig. 2:**
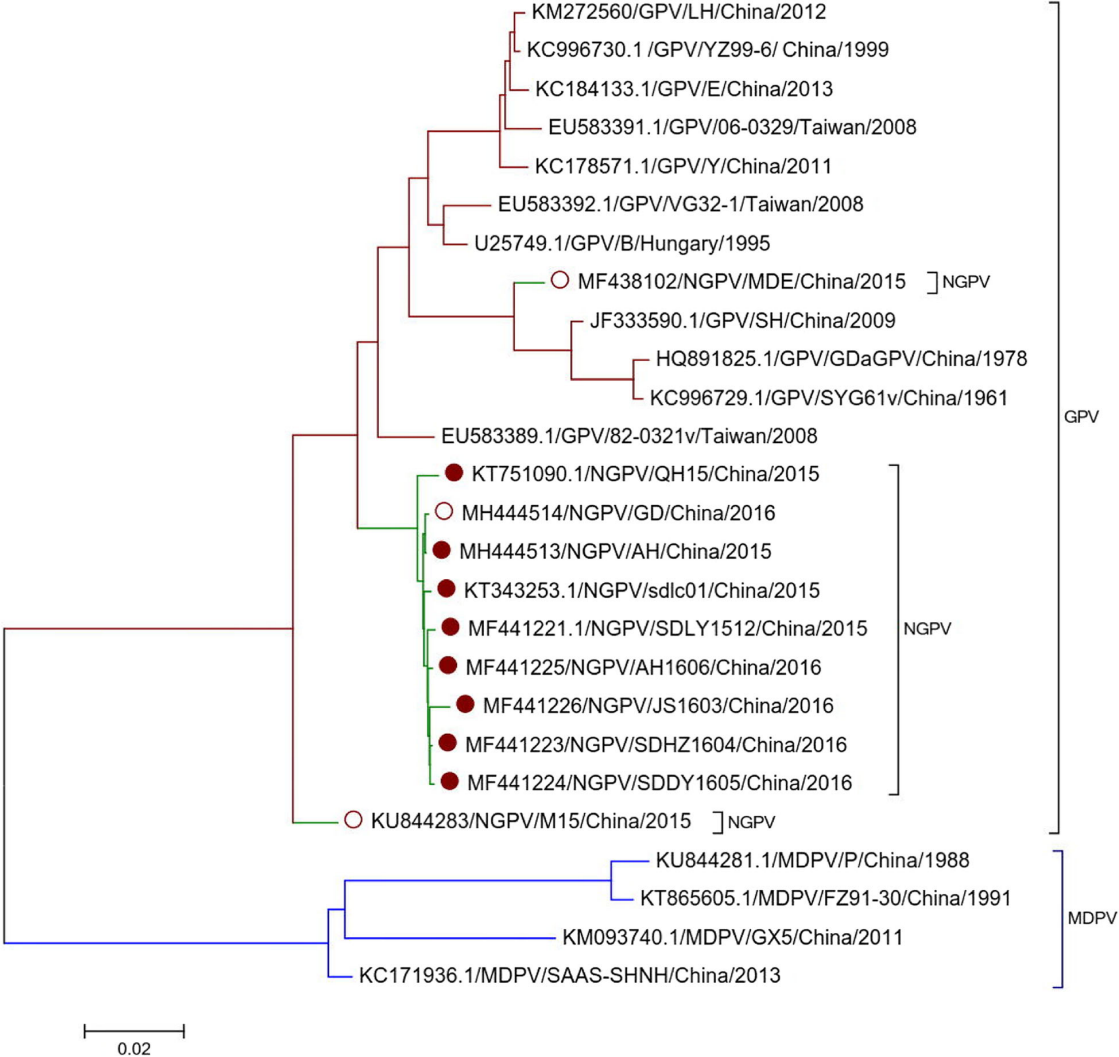
Phylogenetic analysis of GPV, MDPV and NGPV complete genome. Phylogenetic analysis of strain AH and GD with 11 GPV, 4 MDPV, 7 NGPV isolated from Cherry Valley duck (solid circle) and 2 NGPV isolated from mule duck (hollow circle). Those isolates have complete genome sequences deposited in GenBank. The neighbor-joining method in MEGA 5.0 was used for construction of phylogenetic trees

**Fig. 3:**
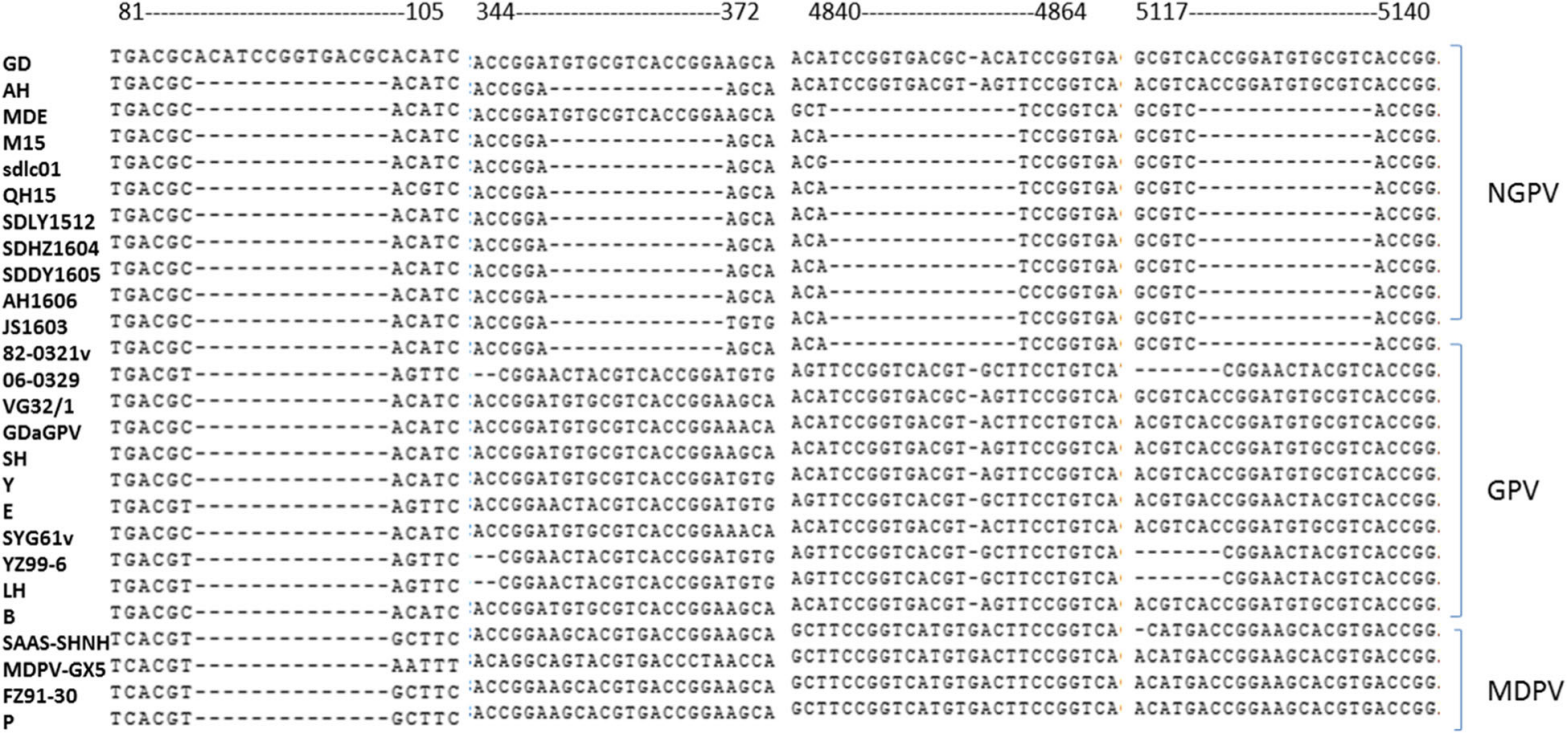
Alignment of nucleotide sequences of GD and AH with other NGVP, GPV and MDPV. Dashes denote nucleotide deletions. The numbers above the alignment denote the nucleotide’s position

Phylogenetic analysis of NGPV, GPV and MDPV sequences revealed that all NGPV isolates have more similarity with GPV than MDPV (Fig. 2). Homology among NGPV isolates ranges from 93.4 to 99.9% at whole genome level. All NGPV isolates share 89.7–96.7% identity with classical GPV and 79.6–83.9% identity with MDPV. These results revealed that there is no significant difference among NGPV isolated either from mule duck or Cherry Valley duck and also the NGPV isolates are more closely related to GPV virus. Sequence alignment of NGPV with classical GPV and MDPV using MEGA 5.0 software revealed three 14-nucleotide sequences deletion in most of NGPV isolates (Fig. 3). A putative recombination was also observed in GD strain between 87 nt – 100 nt (Fig. 3).

### Phylogenetic analysis of NS genes

**Fig. 4:**
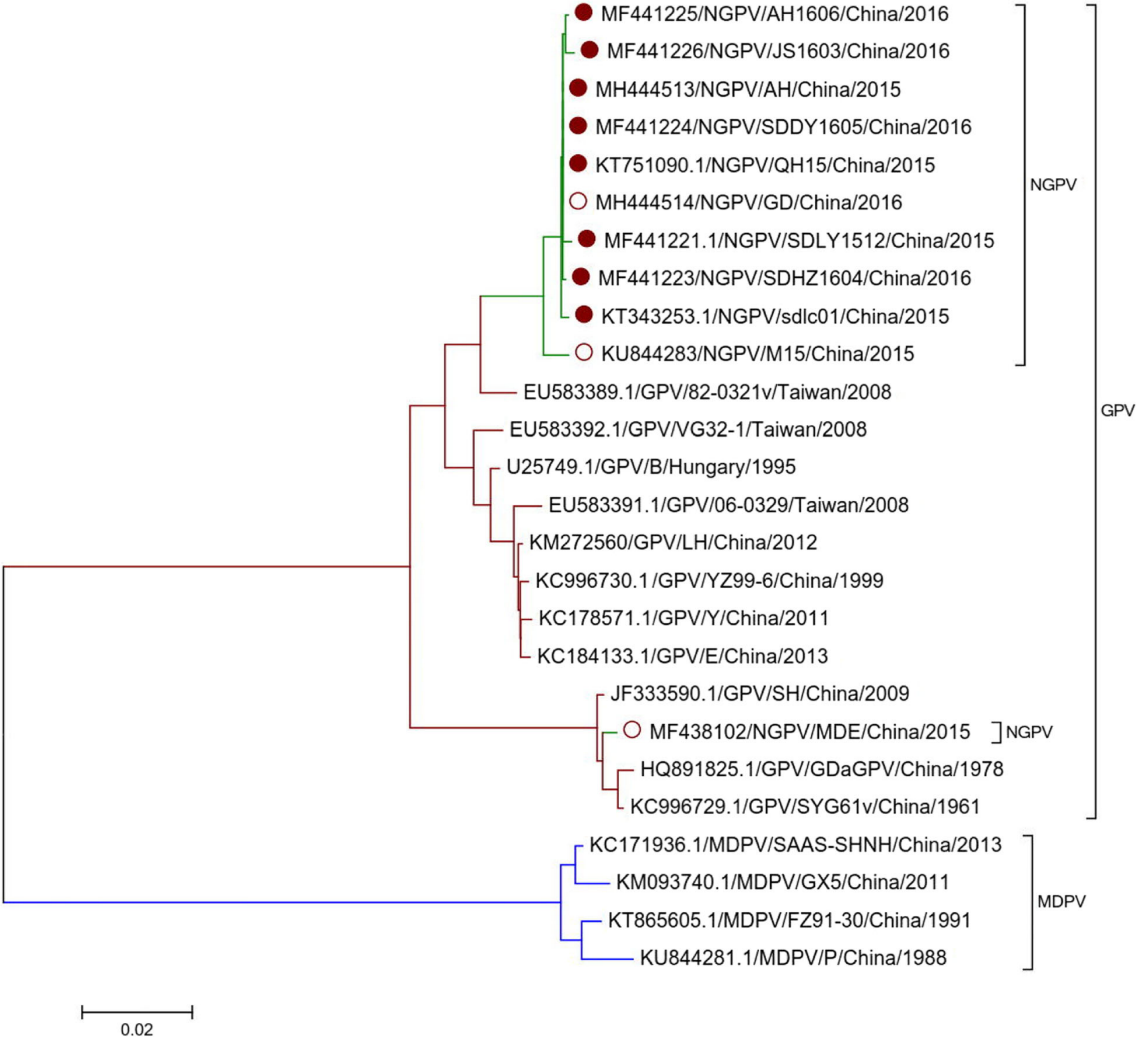
Phylogenetic analysis of GPV, MDPV and NGPV NS genes. Phylogenetic analysis of strain AH and GD with 11 GPV, 4 MDPV, 7 NGPV isolated from Cherry Valley duck (solid circle) and 2 NGPV isolated from mule duck (hollow circle). Those isolates have NS genes sequences deposited in GenBank. The neighbor-joining method in MEGA 5.0 was used for construction of phylogenetic trees

In order to understand the genetic diversity of Non-structural proteins (NS) in NGPV, GPV and MDPV isolates, phylogenetic tree was constructed and homology analysis was also carried out (Fig. 4). Through sequence alignment, it was found that the length of NS proteins of all NGPV, GPV and MDPV sequences is 1881 nt, and they encode 626 amino acids. Through comparison of NS gene, both NGPV isolates (AH and GD) have more similarly with GPV than MDPV, which is similar to complete genome sequence findings.

Homology of NS proteins among NGPV isolates ranged between 93.7–100% at gene level and 95.4–100% at amino acid level. However, they share 93.4–97.9% identity with classical GPV and 81.0–82.4% identity with MDPV. At amino acid level, all NGPV isolates shared 96.3–99.5% identity with classical GPV and 89.0–90.1% identity with MDPV. As compared to classical GPV and MDPV, there are twelve synchronous specific mutations in NS amino acids (Table 3).

**Table 3:** Amino acid mutations in replication

virus	Sites in replication protein											
	50	131	140	350	468	498	553	555	573	594	605	617
NGPV	T	R	S	V	I	R	K	T	Q	Y	T	A
GPV	I	K	A	A	V	E	R	N	E	D	K	V
MDPV	I	K	A	A	V	E	R	D	E	N	N	V

### Phylogenetic analysis of VP genes

**Fig. 5:**
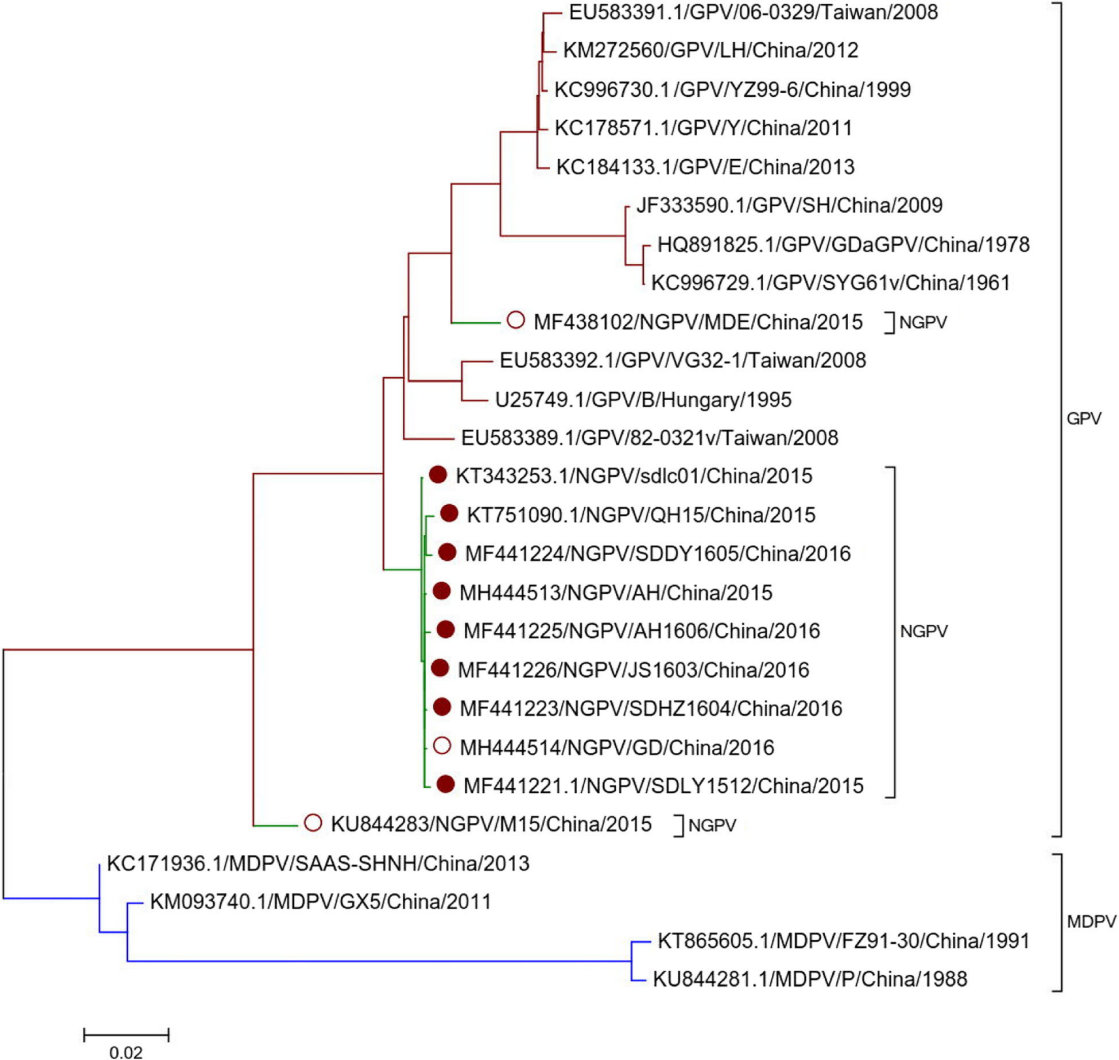
Phylogenetic analysis of GPV, MDPV and NGPV VP gene. Phylogenetic analysis of strain AH and GD with 11 GPV, 4 MDPV, 7 NGPV isolated from Cherry Valley duck (solid circle) and 2 NGPV isolated from mule duck (hollow circle). Those isolates have VP gene sequences deposited in GenBank. The neighbor-joining method in MEGA 5.0 was used for construction of phylogenetic trees

The genetic diversity of VP gene (Capsid protein gene) was also studied by phylogeny and homology analysis (Fig. 5). Through sequence alignment, it was found that the length of all VP genes of NGPV, GPV and MDPV is 2199 nt and they encode 732 amino acids. The comparison of VP gene also showed more similarity of NGPV (AH and GD) sequences with GPV than MDPV sequences, which is similar to the findings of complete genome sequence and NS gene analysis.

Homology among NGPV isolates ranged between 94.0–100% at VP gene level and 96.9–100% at amino acid level. The VP gene of NGPV isolates has 90.9–97.5% identity with classical GPV and 80.9–91.5% identity with MDPV. At VP amino acid level, NGPV isolates shared 95.1–98.2% identity with classical GPV, and 88.0–92.6% identity with MDPV. As compared to classical GPV and MDPV, there are eight synchronous specific mutations in VP amino acids (Table 4).

**Table 4:** Simultaneous amino acid mutations in capsid protein

virus	Sites in capsid protein							
	89	114	116	142	180	450	498	660
NGPV	L	H	H	E	V	N	N	N
GPV	Q	D	Q/R	D	A	S	S	H
MDPV	Q	D	T	D	G	N/G	N/S	N

### Histopathological examination

**Fig. 6:**
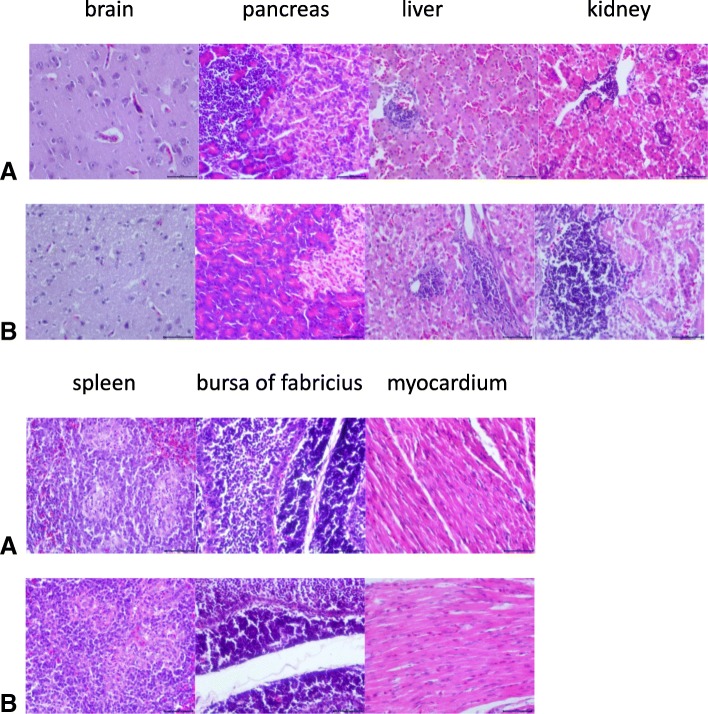
Microscopic lesions in tissues of ducks. A, AH-infected animals; B, uninfected control animals

To find out the damage caused by NGPV to tissues and organs, including kidney, brain, pancreas, liver, spleen, bursa of fabricius and myocardial tissues, histopathological investigations were carried out (Fig. 6). In both experimental and control animals, myocardium and bursa of fabricius are perfectly normal and the other tissues have inflammatory cells exudation. The results suggested that NGPV has no significant influence on these tissues and organs.

## Discussion

In this study, we studied the complete genome sequence of AH and GD, isolated from mule duck and Cherry Valley duck flocks reported with SBDS from Anhui and Guangdong province, China. SBDS in ducks was first reported in France in 1970s [14], followed by outbreaks in Taiwan (1989), Poland (1995) [2, 14], Hungary (2009) [14] and mainland China (2014) [20]. Although the genome sequences of NGPV, GPV and MDPV strains have been reported previously, but this is the first study, which reported variations among various parvovirus duck strains on the basis of phylogeny and homology analysis [4, 14, 17, 21]. The sequence analysis indicated that all NGPV isolates have more homology among them as compared to classical GPV and MDPV. Several studies reported SBDS in ducks maybe caused by novel-MDPV is also reported, but the whole genome sequence analysis has shown that GPV clade also exists in this paper, which the rusults might be due to coinfection with NGPV and MDPV strains [22].

The complete genome sequence of AH and GD strains contain 5053 nt and 5106 nt respectively and they shared 93.4–99.9% identity with other NGPV, 89.7–96.7% identity with classical GPV and 79.6–83.9% identity with MDPV strains. The length of ITRs of AH and GD is 379 and 407 nt respectively. Through sequence alignment, it was found that the length of NS protein of all NGPV, GPV and MDPV strains is 1881 nt encoding 626 amino acids and the length of VP gene of all NGPV, GPV and MDPV strains is 2199 nt which encodes 732 amino acids. When comparison was carried out between mule duck isolates including GD and Cherry Valley duck isolates including AH, it was found that NGPV isolated from mule duck and those isolated from Cherry Valley duck have no significant differences among them.

VP protein plays an important role in the stimulation of protective response in the body against the virus as it contains viral antigenic sites [23–25]. Interestingly VP protein of NGPV and classical GPV shares 90.9 to 97.5% homology, therefore it is very likely that vaccine against GPV is effective against NGPV. However, in this case, it’s protective efficacy may slightly decrease. Further studies are required to explore the effectiveness of currently available vaccines against NGPV and to develop a new vaccine for NGPV. It will be a difficult task as virus titer is low in duck embryo and large scale culturing of NGPV would be challenging.

The complete genome sequence analysis also showed that most of NGPV isolates have several 14-nucleotide sequence deletions not found in classical GPV and MDPV sequences. It was also noticed and reported for the first time that the 14-nucleotide sequences deletion is identical to each other and the front and back of deleted position also have a same 14-nucleotide sequences. These results lead us to suspect that the 14-nucleotide sequences deletion play a major role in migration of host, if sequencing mistakes are excluded. The SimPlot analysis of GD strain with other strains revealed no clear indication of increase in genome at at 87 nt − 100 nt. However, as it is too short (14 nt), therefore it might be possible that SimPlot software could not able to use analysis recombination.

The histopathological examination revealed no remarkable difference in kidney, brain, pancreas, liver, spleen, bursa of fabricius, myocardium tissues between experimental group and control group. These findings suggested that NGPV causes non-significant pathological changes in these organs and tissues, which might be the reason of low mortality in infected animals (2%).

In conclusion, the novel parvovirus-related virus is responsible for SBDS disease outbreaks in eastern China since 2014 [21, 26]. The low mortality and smaller beak is the major difference between NGPV and GPV infected animals. NGPV is hard to culture in duck embryos and DEF (duck embryos fibroblast). Since classical GPV does not infect mule duck and Cherry Valley ducks, therefore NGPV is not completely adapted to cause severe disease in mule duck and Cherry Valley duck which might be the reason for low mortality caused by these strains. The NGPV also has little pathological impact on kidney, brain and other organs and tissues. However, further studies are required to evaluate the protective efficacy of classical GPV vaccine against SBDS and to develop a new vaccine for this disease.

## Conclusions

AH and GD strains isolated from mule duck and Cherry Valley duck flocks are novel parvovirus-related virus. The comparison of complete genome sequence of eleven NGPV isolates from mule ducks and cherry valley ducks found no remarkable difference between them. Through histopathological examination, it was found that both experimental and control groups have perfectly normal myocardium and bursa of fabricius while other tissues have inflammatory cells exudation. The results suggested that NGPV has no significant influence on the tissues and organs. This study has contributed better understanding of molecular biology of NGPV strains and will help to develop the candidate strain for vaccine preparation in future.

## Abbreviations

GPV: Goose parvovirus
ITR: Inverted terminal repeats
Kb: Kilobase
MDPV: Muscovy duck parvovirus
NGPV: Novel parvovirus-related virus
NS: Non-structural proteins
Nt: Nucleotides
ORF: Open reading frame
SBDS: Short beak and dwarfism syndrome
VP: Capsid protein gene

## References

[1] Glávits R, Zolnai A, Szabó E, Ivanics E, Zarka P, Mató T, Palya V (2005). Comparative pathological studies on domestic geese (Anser anser domestica) and Muscovy ducks (Cairina moschata) experimentally infected with parvovirus strains of goose and Muscovy duck origin. Acta Vet Hung.

[2] Woźniakowski G, Kozdruń W, Samoreksalamonowicz E (2009). Genetic variance of Derzsy's disease strains isolated in Poland. Journal of Molecular & Genetic Medicine An International Journal of Biomedical Research.

[3] Derzsy D, Kisary J. Viral disease of goslings: World Veterinary Congress; 1975.

[4] Yu K, Ma X, Sheng Z, Qi L, Liu C, Dan W, Bing H, Feng L, Song M (2016). Identification of goose-origin parvovirus as a cause of newly emerging beak atrophy and dwarfism syndrome in ducklings. J Clin Microbiol.

[5] Kisary J: Some growth characteristics of goose parvovirus strain "B**"**. Acta Vet Acad Sci Hung 1974, 24(3):329. 4456997

[6] Takehara K, Hyakutake K, Imamura T, Mutoh KI, Yoshimura M (1994). Isolation, identification, and plaque titration of parvovirus from Muscovy ducks in Japan. Avian Dis.

[7] Le GRG, Jestin V (1994). Biochemical and genomic characterization of Muscovy duck parvovirus. Arch Virol.

[8] Wang J, Duan J, Meng X, Gong J, Jiang Z, Zhu G (2014). Cloning of the genome of a goose parvovirus vaccine strain SYG61v and rescue of infectious virions from recombinant plasmid in embryonated goose eggs. J Virol Methods.

[9] Wang J, Huang Y, Zhou M, Hardwidge PR, Zhu G (2016). Construction and sequencing of an infectious clone of the goose embryo-adapted Muscovy duck parvovirus vaccine strain FZ91-30. Virol J.

[10] Ji J, Xie QM, Chen CY, Bai SW, Zou LS, Zuo KJ, Cao YC, Xue CY, Ma JY, Bi YZ (2010). Molecular detection of Muscovy duck parvovirus by loop-mediated isothermal amplification assay. Poult Sci.

[11] Shen H, Zhang W, Wang H, Zhou Y, Shao S (2015). Identification of recombination between Muscovy duck parvovirus and goose parvovirus structural protein genes. Arch Virol.

[12] Zádori Z, Stefancsik R, Rauch T, Kisary J (1995). Analysis of the complete nucleotide sequences of goose and Muscovy duck Pervoviruses indicates common ancestral origin with adeno-associated virus 2. Virology.

[13] Poonia B, Dunn PA, Lu H, Jarosinski KW, Schat KA (2006). Isolation and molecular characterization of a new Muscovy duck parvovirus from Muscovy ducks in the USA. Avian Pathology.

[14] Palya V, Zolnai A, Benyeda Z, Kovács E, Kardi V, Mató T (2009). Short beak and dwarfism syndrome of mule duck is caused by a distinct lineage of goose parvovirus. Avian Pathology.

[15] Villate D, Caldier P (1989). Manuel pratique des maladies des palmipedes.

[16] Chen H, Dou Y, Tang Y, Zhang Z, Zheng X, Niu X, Yang J, Yu X, Diao Y (2015). Isolation and genomic characterization of a duck-origin GPV-related parvovirus from Cherry Valley ducklings in China. PLoS One.

[17] Li P, Lin S, Zhang R, Chen J, Sun D, Lan J, Song S, Xie Z, Jiang S. Isolation and characterization of novel goose parvovirus-related virus reveal the evolution of waterfowl parvovirus. Transboundary & Emerging Diseases. 2017;65(2). 10.1111/tbed.12751 29143488

[18] Fan W, Sun Z, Shen T, Xu D, Huang K, Zhou J, Song S, Yan L (2017). Analysis of evolutionary processes of species jump in waterfowl parvovirus. Front Microbiol.

[19] Tamura K (2013). MEGA5: molecular evolutionary genetics analysis version 6.0. Molecular Biology & Evolution.

[20] Chen S, Wang S, Cheng X, Xiao S, Zhu X, Lin F, Wu N, Wang J, Huang M, Zheng M (2016). Isolation and characterization of a distinct duck-origin goose parvovirus causing an outbreak of duckling short beak and dwarfism syndrome in China. Arch Virol.

[21] Xiao S, Chen S, Cheng X, Lin F, Wang S, Zhu X, Yu B, Huang M, Wang J, Wu N (2017). The newly emerging duck-origin goose parvovirus in China exhibits a wide range of pathogenicity to main domesticated waterfowl. Vet Microbiol.

[22] Fu Q, Huang Y, Wan C, Fu G, Qi B, Cheng L, Shi S, Chen H, Liu R, Chen Z (2017). Genomic and pathogenic analysis of a Muscovy duck parvovirus strain causing short beak and dwarfism syndrome without tongue protrusion. Res Vet Sci.

[23] Berns KI (1990). Parvovirus replication. Microbiol Rev.

[24] Salganik M, Aydemir F, Nam HJ, Mckenna R, Agbandjemckenna M, Muzyczka N (2014). Adeno-associated virus capsid proteins may play a role in transcription and second-Strand synthesis of recombinant genomes. J Virol.

[25] Hueffer K, Parker JSL, Weichert WS, Geisel RE, Sgro JY, Parrish CR (2003). The natural host range shift and subsequent evolution of canine parvovirus resulted from virus-specific binding to the canine transferrin receptor. J Virol.

[26] Ning K, Wang M, Qu S, Lv J, Yang L, Zhang D (2017). Pathogenicity of Pekin duck- and goose-origin parvoviruses in Pekin ducklings. Vet Microbiol.

